# The Staining Susceptibility and Surface Roughness of Teeth Restored by Microabrasion and Resin Infiltration: An In Vitro Study

**DOI:** 10.3390/polym16243523

**Published:** 2024-12-18

**Authors:** Treetossatep Inna, Nantawan Krajangta, Thanasak Rakmanee

**Affiliations:** Department of Restorative Dentistry, Faculty of Dentistry, Thammasat University, 99 M. 18, Paholyothin Road, Klong Luang, Pathum Thani 12120, Thailand; treetossatepi@gmail.com (T.I.); knantawa@gmail.com (N.K.)

**Keywords:** resin infiltration, microabrasion, staining, surface roughness, thermocycling, spectrophotometer

## Abstract

This study assessed the susceptibility to staining and surface roughness of white-spot lesions (WSLs) treated with resin infiltration (RIT) and microabrasion (MA) under simulated aging through thermocycling in red wine. Seventy-eight extracted human premolars with artificial WSLs were divided into three groups: untreated WSLs (control), RIT-treated (ICON^®^, DMG), and MA-treated (Opalustre^®^, Ultradent). Each group was further split: one subgroup immersed in artificial saliva and the other thermocycled in red wine. The color change (∆E) and surface roughness (R_a_) were measured before and after staining using a spectrophotometer and a non-contact profilometer. Thermocycling in red wine increased color change (ΔE) across all groups, with the highest values observed for MA (43.94 ± 3.57), followed by RIT (31.40 ± 4.89). Surface roughness (R_a_) was highest in untreated WSLs (0.61 ± 0.18 µm) and lowest in RIT (0.15 ± 0.03 µm). While RIT and MA similarly improved WSL appearance, RIT exhibited superior smoothness. These findings suggest that RIT provides a more durable surface with reduced roughness, although staining susceptibility remains comparable to MA. Moderate positive correlation was found between ΔE and Ra, indicating that roughness is one of the factors influencing color changes.

## 1. Introduction

Dental caries are a prevalent oral disease caused by the metabolic activity of bacteria, which results in the demineralization of tooth structure through acid production. The earliest clinical manifestation of this process is the appearance of white-spot lesions (WSLs), characterized by white opaque areas on the enamel surface due to subsurface mineral loss [1]. WSLs not only indicate the potential for progression to cavitated caries but also pose significant esthetic concerns, especially when located in visible areas during smiling or speaking. The esthetic impact of WSLs arises from the contrast in refractive indices (RIs) between healthy enamel (RI = 1.62) and the microporosities in the lesion, filled with water (RI = 1.33) or air (RI = 1.0). This disparity causes light scattering, giving WSLs their characteristic chalky appearance [2].

Management of WSLs has evolved from traditional restorative approaches to minimally invasive techniques that focus on prevention and early intervention. When WSLs are in a reversible stage, treatment aims to balance remineralization and demineralization. Promoting remineralization involves guiding patients’ oral hygiene care, regulating sugar consumption, and using agents such as fluoride or casein phosphopeptide—amorphous calcium phosphate (CPP-ACP) [1]. These agents can slow lesion progression by enhancing remineralization, but they often fail to improve esthetic outcomes immediately [2], as remineralization tends to be superficial [3], leaving the subsurface porosities and whitish appearance intact [4].

Resin infiltration (RIT) (ICON^®^) is a technique that uses low-viscosity resin to penetrate the porous enamel caries [5,6,7]. Once infiltrated, the resin’s RI (~1.46) closely matches that of sound enamel, improving the lesion’s appearance [2]. Microabrasion (MA) is another minimally invasive treatment involving mechanical abrasion and acid erosion to create a shiny, lustrous enamel surface [8]. However, excessive abrasion can result in enamel loss and tooth sensitivity. While MA has been considered easy to perform, time efficient, and cost effective [8], RIT is regarded as more conservative in terms of enamel preservation [8,9].

A recent study on the treatment of white-spot lesions (WSLs) using RIT and MA have shown that both techniques effectively enhance the esthetic appearance of WSLs, with RIT being more effective in managing WSLs [10,11]. Both treatments can maintain color stability within a short periods of 3 months [10], and patients are satisfied with both treatment results after 6 months [11]. Additionally, current treatments incorporate microabrasion combined with resin infiltration to manage WSLs, yielding good outcomes [12].

The oral environment is subject to thermal stress and pH fluctuations from food and beverage consumption, which can impact the surface properties of resin materials, leading to long-term discoloration [13]. Over time, direct dental restorations, particularly resin composites, may exhibit surface wear and filler–matrix debonding, resulting in void formation [14]. In laboratory settings, the aging of dental material is typically evaluated through factors such as increased water sorption, surface roughness, and color changes, along with reduced strength and hardness [13,15]. Thermocycling is widely used to simulate the physical aging of dental materials by repeatedly subjecting them to alternating hot and cold water baths, rather than relying solely on intraoral thermal variations [16]. This present study has been further refined through the use of thermocycling stain models, which provide a more comprehensive simulation of the oral environment. Such models account for the thermal fluctuations, time-dependent interactions, and exposure to staining agents commonly found in beverages [17]. Despite these advancements, no existing study has investigated the dynamic staining susceptibility of RIT and MA when exposed to thermocycling conditions using staining solutions.

Staining susceptibility is a critical factor affecting the longevity of esthetic outcomes. Among the various factors influencing discoloration [18], surface roughness (R_a_) plays a notable role, as it can increase the material’s susceptibility to staining, particularly from external sources like food and beverages [19]. Several studies investigated the staining susceptibility of RIT and MA, finding that both treatments may undergo color changes when exposed to staining solutions [20,21,22,23,24]. Most studies on the staining susceptibility of RIT [21,22,23,24,25,26] and MA [20,23] use static models, where samples are immersed in staining solutions to measure color changes. However, these static models may not replicate the dynamic and multifactorial conditions of the oral environment, including thermal stress, acidic pH, and fluid interactions.

This present study addresses these gaps by evaluating the dynamic staining susceptibility and surface roughness of RIT and MA under thermocycling in red wine, a highly staining-prone agent. This approach integrates thermal stress to simulate real-life oral conditions, enhancing the generalizability and clinical relevance of the findings. By adopting this systematic and comprehensive methodology, this research aims to provide a more nuanced understanding of the esthetic longevity of RIT and MA, ultimately guiding clinicians in making evidence-based decisions for managing WSLs.

## 2. Materials and Methods

This study aimed to compare the staining susceptibility and surface roughness of teeth treated with RIT and MA under thermal stress induced by thermocycling in red wine. The null hypotheses are as follows: (1) no statistical difference in staining susceptibility between RIT- and MA-treated teeth after thermocycling in red wine; (2) no statistical difference in surface roughness between resin-infiltrated and micro-abraded teeth after thermocycling in red wine; and (3) no statistical correlation between staining susceptibility and surface roughness.

This in vitro study utilized extracted human permanent premolar teeth, obtained for orthodontic purposes and selected based on their color within the A3–A4 range using a spectrophotometer (VITA Easyshade^®^ V from VITA Zahnfabrik H. Rauter GmbH & Co. KG. Bad Säckingen, Germany). Ethical approval was obtained from The Human Research Ethics Committee of Thammasat University (Science) under project number 66DE140.

### 2.1. Experimental Design

**Figure 1:**
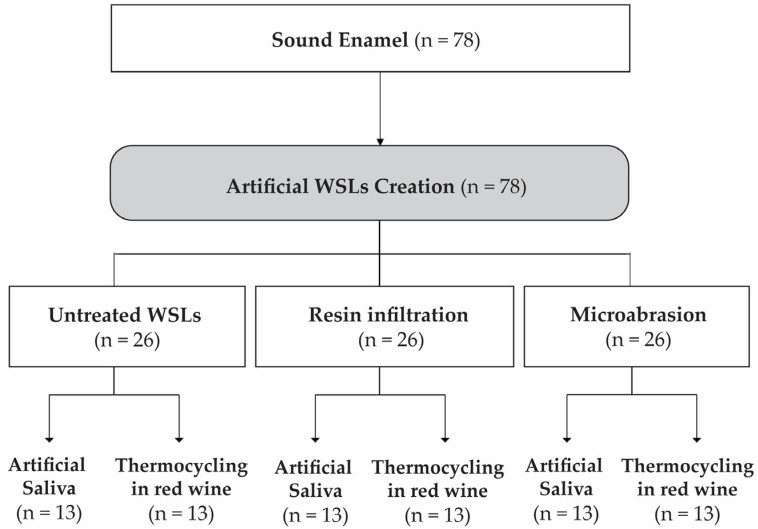
Flow diagram of the experimental procedure.

The study design is summarized in Figure 1. The baseline color and surface roughness (R_a_) of all sound enamel samples were recorded. To create artificial WSLs, the samples were immersed in a demineralization solution. A total of 78 WSLs were created and equally divided into three groups: untreated WSLs, resin infiltration (RIT), and microabrasion (MA). The samples were kept at room temperature, dried, and shielded from direct sunlight. Each group was further subdivided into two subgroups: one immersed in artificial saliva as a control, and the other subjected to thermocycling in red wine. The color and R_a_ were measured at each experimental stage, as described below.

### 2.2. Specimen Preparation

A sample size of 78 extracted permanent premolars were calculated using GPower version 3.1.9.7, with a significance level of 0.05 and a power of 0.95 (standard deviation of means from a pilot study: 5.63, unpublished data). The teeth were cleaned with an ultrasonic cleaner and a non-fluoride pumice paste and then stored in 0.1% thymol at 4 °C to disinfect and maintain their structural integrity. Each tooth was sectioned at the cementoenamel junction to remove the root, preserving the crown.

**Figure 2:**
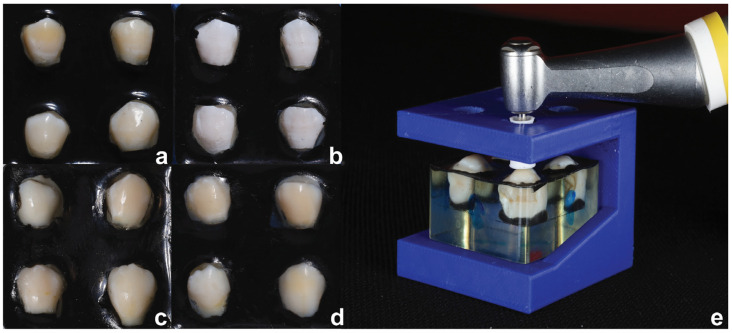
The specimens in each treatment: (**a**) sound enamel, (**b**) artificial white-spot lesion (**c**) after resin infiltration, (**d**) after microabrasion, and (**e**) 3D printing template designed for microabrasion.

The crowns were mounted in acrylic blocks (Figure 2) with the buccal surface exposed (~5 × 5 mm^2^), protected by black nail varnish [27]. The specimens were immersed in a demineralization solution, as described by Yuan et al. (2014), comprising 6% hydroxylethyl cellulose and 0.1 M lactic acid, at pH 4.5, and at 37 °C for one week to create WSLs [28]. Following demineralization, specimens were rinsed with distilled deionized water for 30 s, and WSL formation was visually inspected and confirmed by a spectrophotometer (VITA Easyshade^®^ V).

#### 2.2.1. Resin Infiltration (RI) Group

The WSLs in this group were treated with RIT (ICON^®^ infiltrant; DMG, Hamburg, Germany) following the manufacturer’s instructions. The lesion surface was etched with 15% hydrochloric acid (HCL) (ICON^®^ etch) for 2 min, rinsed with a water spray for 30 s, and dried. Then, 99% ethanol (ICON^®^ dry) was applied for 30 s. A low-viscosity resin (ICON^®^ infiltrant) was applied for 3 min and light cured for 40 s (SmartLite^®^ Focus^®^, Dentsply Sirona, Bangkok, Thailand). A second resin layer was applied for 1 min and light cured. The samples were polished using Al_2_O_3_ disks (Sof-Lex Pop on, 3M-ESPE, St. Paul, MN, USA) from fine to superfine for 30 s [29].

#### 2.2.2. Microabrasion (MA) Group

The WSLs in this group were treated with MA (Opalustre^®^; Ultradent, South Jordan, UT, USA), containing 6.6% HCL and silicon carbide particles (20–160 µm). The slurry was applied using OpalCups^®^ attached to a low-speed handpiece (NSK-EC^®^; Nakanishi Inc., Tokyo, Japan), with light pressure for 1 min, with three repeated applications (Figure 2d) [20]. After each application, the enamel surface was rinsed with a water spray for 10 s. A 3D-printed template ensured perpendicular application of OpalCups^®^ (Figure 2e). The surface was polished with Al_2_O_3_ disks, as described above [29] and treated with 2% sodium fluoride gel (Pro-F^®^, Prominent, Bangkok, Thailand) for 4 min [20], followed by a 30 s rinse.

### 2.3. Thermocycling in Staining Solution

Each experimental group was divided into control (non-exposed, immersed in artificial saliva) and staining subgroups subjected to thermocycling in red wine [17]. Control samples were kept in artificial saliva (methylcellulose, glycerin, paraben, and purified water) at room temperature. The staining subgroup underwent thermocycling between red wine baths (Nadin Bin777, Cabernet Sauvignon, South Australia, pH = 3.53) at 5 °C and 55 °C, for 30 s each. A pH meter was used to ensure the solution’s pH remained stable. Thermocycling (5000 cycles) simulated six months of aging (HWB332R, TC301, King Mongkut’s Institute of Technology, Bangkok, Thailand). Color and R_a_ were then measured after thermocycling.

### 2.4. Measurement of Staining Susceptibility

**Figure 3:**
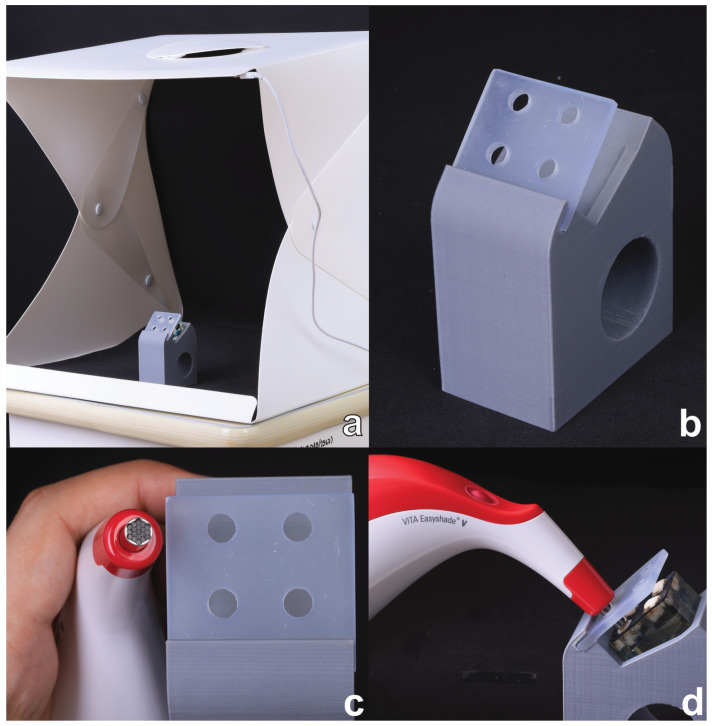
A 3D-printed resin model and color measurement protocols. (**a**) Viewing box with a D65 illuminant. (**b**) A 3D-printed model. (**c**) The template was designed to be the same size as the tip of the VITA Easyshade^®^ V. (**d**) The 45°/0° standard measurement was controlled by the 3D-printed model.

Color changes were analyzed using the CIEDE2000 system (∆E_00_), based on the formula by Sharma et al. (2005) [30]. Measurements were performed using a spectrophotometer (VITA Easyshade^®^ V), with each sample air-blown for 30 s prior to measurement. All color measurements were conducted under standardized conditions according to ISO/TR 28642-2016 [31], using a viewing box with a D65 illuminant (representing noon daylight) and a controlled illuminance of 1000 lux measured by a lux meter. A 45°/0° optical geometric observation was used for assessment [31,32], with sample positioning standardized by a 3D-printed model to ensure accuracy and reproducibility (Figure 3).

### 2.5. Surface Roughness Measurement

R_a_ was assessed using a non-contact profilometer (InfiniteFocus G5, Alicona, Raaba/Graz, Austria), at the mid-buccal surface of the enamel. Measurements were taken at the same position as the baseline.

### 2.6. Statistical Analysis

Data were analyzed using the SPSS 26.0 (IBM, Chicago, IL, USA) software. Normality was assessed using the Kolmogorov–Smirnov test. Median and median absolute deviation (MAD) described nonparametric data. Wilcoxon signed-rank and Friedman tests were employed for intra-group comparisons, while Kruskal–Wallis’s tests were evaluated for inter-group differences in staining susceptibility and R_a_. Post hoc Bonferroni correction identified significant differences between paired groups. Spearman correlation analysis examined the relationship between staining susceptibility and R_a_. A significant level of 0.05 was used for all tests.

## 3. Results and Discussion

### 3.1. Color Values (L*, a*, b*)

Descriptive statistics for the color parameters (L*, a*, and b*) are presented in Table 1. Significant differences were observed in all color parameters after the creation of artificial WSLs compared to sound enamel (*p* < 0.05). The L* value, indicating brightness, significantly decreased after WSL formation, reflecting a loss of brightness. The a* value (red–green) increased significantly, while the b* value (yellow–blue) slightly decreased, signaling a shift in color.

Post-treatment with RIT and MA, the color parameters approached those of sound enamel. In the RIT group, L* increased significantly, restoring it closer to the original enamel value. The a* and b* values also shifted towards the baseline, indicating improved color consistency. In the MA group, L* values significantly increased compared to WSL, with similar improvements in a* and b* values. However, the b* value in the RIT remained lower than in sound enamel, reflecting some residual differences in the yellow–blue spectrum.

Post-treatments with RIT and MA, all color parameters returned close to sound enamel, except for b* value in RIT, which might have been due to the slight difference in the refractive index between resin infiltration (1.46) and sound enamel (1.62), which may explain the b* value’s persistence [2]. These results suggest that both procedures effectively treat WSLs.

### 3.2. Surface Roughness (R_a_)

Changes in R_a_ throughout the experiment are summarized in Table 2. Following WSL formation, R_a_ increased in all groups compared to baseline, with significant differences observed in groups 1, 2, 4, and 5 (*p* < 0.05). Both RIT and MA treatments reduced surface roughness compared to untreated WSLs. Significant differences were noted in groups 3, 5, and 6 (*p* < 0.05). All groups exhibited a decrease in R_a_ after immersion in artificial saliva (control), with a significant difference observed in groups 2 and 6.

Following thermocycling in red wine, the untreated WSL and MA groups experienced substantial increases in R_a_. Conversely, R_a_ in the RIT group modestly decreased, although this change was not statistically significant compared to baseline.

Surface roughness (R_a_) is a key parameter for evaluating surface texture and can change over time. In this study, R_a_ was measured using the InfiniteFocus G5 optical profilometer, which avoids surface contact, preventing damage and measurement errors associated with stylus tip devices [33]. The polishing protocol used significantly influenced R_a_ values; both RIT and MA followed the same polishing protocol. Previous research shows that Al_2_O_3_ disks (Sof-Lex Pop on) effectively reduced R_a_ in resin-infiltrated enamel compared to silicon carbide tips and brushes [29]. While polishing after RIT may not reduce bacterial adhesion, it remains crucial for minimizing the risk of staining [29].

After WSLs were created, the R_a_ of the samples increased compared to the baseline (Table 2). The lack of significant differences in R_a_ between groups 3 and 6 may be due to the small sample size, limiting the detection of statistical significance. Larger sample sizes in future studies may help address this limitation. Consistent with previous research, we observed that the R_a_ of WSLs was higher than that of sound enamel [34,35,36]. Both RIT and MA reduced surface roughness, similar to findings by Yazkan and Ermis (2018), who reported lower R_a_ with RIT than MA [36]. However, in our study, R_a_ for RIT was slightly higher than MA, likely due to variations in the MA protocol. While Yazkan and Ermis used 30 s Opalustre^®^ polishing repeated in two cycles with 2–4 µm diamond paste, our study employed a more intensive protocol—1 min polishing, repeated in three cycles with 24 µm (fine) to 8 µm (superfine) Al_2_O_3_ disks [20,37]. Despite this, both treatments produced surface roughness values similar to sound enamel.

After thermocycling in a staining model, the R_a_ of the RIT group trended to increase compared to the control group, although this difference was not statistically significant. Thermal stress from thermocycling may impact the surface integrity of resin-infiltrated enamel lesions, potentially creating microcracks and increasing roughness [24]. This study also showed that R_a_ in the RIT group was lower than in the MA group, although the difference was not significant, leading to the acceptance of the second null hypothesis. No prior studies have specifically investigated the surface roughness of RIT and MA after thermocycling in red wine staining. In addition to thermal stress, the erosive effect of red wine likely plays a significant role in altering the enamel surface after MA.

### 3.3. Color Changes (∆E)

Color changes (ΔE) are presented in Table 3. In the control group (artificial saliva), untreated WSLs exhibited a moderate color change (median ΔE of 2.77). RIT and MA treatments showed lower ΔE values (1.38 and 1.95, respectively) than untreated WSLs, indicating better color stability.

In the staining group exposed to red wine thermocycling, color changes were more pronounced. The untreated WSL group did not provide a measurable ΔE due to extreme discoloration. The RIT group had a median ΔE of 31.40, while the MA group exhibited a higher ΔE of 43.94, indicating significant discoloration in both groups. RIT demonstrated slightly better resistance to red wine staining than MA.

The results showed no significant difference in color change between treatments post-thermocycling, leading to the acceptance of the first null hypothesis. The thermocycling in the staining solution simulated the combined effects of thermal stress, acidic pH, and fluid dynamics from beverage consumption [17]. Previous studies confirmed that resin-based materials are prone to discoloration under these factors [14,38]. Red wine, known for its high staining potential due to tannins, alcohol, and a low pH, surpasses beverages like coffee and tea [39]. The tannins in Cabernet sauvignon grapes penetrate resin materials, contributing to significant staining due to the combined effects of alcohol and a low pH [39,40].

In this study, color change was analyzed using the CIEDE2000 system, which more accurately reflects human color perception than the CIELAB formula [41]. The VITA Easyshade^®^ spectrophotometer used in this study provides accurate and reproducible measurements [42]. However, its limited wavelength range (400–700 nm) likely explains why the purplish discoloration (which occurs in the 385–425 nm range) of untreated WSLs was not detected after staining [43,44]. Despite this, both RIT and MA treatments showed marked improvement in color, with RIT displaying slightly better color stability than MA. Silva et al. (2018) found similar results, indicating no significant difference in color stability between RIT and MA after 7 days of coffee exposure [45]. This supports our finding that both treatments experienced color instability after immersion in red wine. A short-term study also reported that MA was more prone to discoloration than RIT, particularly after 24 h immersion in tea and citric acid [23]. The shorter MA protocol may not fully remove porous enamel, leading to greater discoloration. By contrast, a more extensive MA protocol, while effective, showed that RIT still demonstrated better overall color stability, consistent with Lee et al. (2016) [46]. Color stability may be more affected by dietary staining agents than by the treatment type [47].

The discoloration in RIT-treated teeth arises from both intrinsic and extrinsic factors [24]. Intrinsically, RIT materials contain triethylene glycol dimethacrylate (TEGDMA), a low-viscosity, hydrophilic component, which increases water absorption, allowing for pigment penetration and resulting in color changes [7]. Thermal stress from thermocycling can also cause microcracks and micro-fissures, facilitating pigment infiltration [24]. Extrinsically, pigments from food and beverages adhere to the surface but can be mitigated through polishing, which helps reduce surface staining [24].

MA removes enamel porosities through mechanical abrasion and acid erosion and has been widely used to treat WSLs [8]. For example, Opalustre^®^ has been found to be more effective than Prema^®^ in reducing fluorosis-related WSLs [48]. This present study used a post-treatment fluoride polishing protocol based on Bahadir et al. (2022) [20], which improved stain resistance. Despite this, both RIT and MA treatments showed extreme color changes after dynamic staining, although patient satisfaction with both treatments remains high [11].

The CIEDE2000 color change threshold (∆E_00_) for 50:50% perceptibility is 0.8, and the acceptability threshold is 1.8 [27]. In this study, all groups exceeded the acceptability threshold, except for the RIT group immersed in artificial saliva. Despite the control groups being in artificial saliva, both untreated WSLs and MA exhibited unacceptable color differences. This may be due to differences in measurement protocols. After immersion, the specimens were air-dried for only 30 s, potentially leaving them slightly moist, which could have affected the color measurements [49]. Thus, these variables should be considered for better control in further experiments.

### 3.4. Correlation Between Surface Roughness (R_a_) and Color Change (ΔE)

A significant correlation was found between R_a_ and ΔE (Spearman rank correlation coefficient, r_s_ = 0.577, *p* < 0.00), indicating that changes in surface roughness are moderately positively related to the extent of color change. The findings of this study align with those of previous studies [50,51]. Chowdhury et al. (2021) reported that there was a positive correlation between surface roughness and color changes in nanohybrid composite resin when immersed in artificial saliva, tea, coffee, and Coca-Cola [50]. Additionally, their research demonstrated that both color stability and surface roughness are time dependent, with increased immersion time resulting in greater roughness and more pronounced color changes [50,51]. In this present study, the Spearman correlation test showed linear relationship between staining susceptibility (∆E) and R_a_ for either RIT or MA, leading to the rejection of the third null hypothesis. 

While surface roughness contributes to external staining of dental restorations, it is not the only factor. Other variables, such as water sorption, resin matrix composition, the degree of conversion, polymerization depth, staining agents, and the pH of staining solutions, also affect the discoloration of restorative materials [52].

This study offers valuable insights into the staining susceptibility and surface roughness of RIT and MA, especially for frequent red wine consumers like wine tasters. However, as an in vitro study, it has limitations. The red wine thermocycling model simulates thermal stress, acidic pH, and fluid dynamics [17] but may reflect an extreme discoloration scenario. With 5000 thermocycles simulating six months of aging, no significant difference between both treatments was observed. Future studies with varying thermocycle numbers may reveal differences.

While both RIT and MA effectively restored WSL color to resemble sound enamel, this study did not assess enamel loss, which is critical for determining the best minimally invasive treatment. Additionally, extrinsic staining can be addressed by various methods [53], and further research is also needed to investigate the effectiveness of different polishing or cleaning protocols for reducing stains after RIT and MA.

## 4. Conclusions

Within the limitations of this study, based on the methodology used in thermocycling in the staining solution model, the following can be concluded:

1. After treatment of WSLs, the color parameters (L*, a*, b* values) of both RIT and MA were restored to levels comparable to sound enamel, except for the b* value in the RIT group. This exception may be attributed to a slight difference in the refractive index between resin infiltration (1.46) and sound enamel (1.62), resulting in the persistence of the b* value. These findings suggest that both RIT and MA are effective treatments for WSLs.
2. After thermocycling in red wine, both treatments exhibited extreme discoloration, with median ΔE values of 31.40 ± 4.89 for RIT and 43.94 ± 3.57 for MA. Although the surface roughness of RIT (0.15 ± 0.03 µm) was lower than that of MA (0.55 ± 0.09 µm), the difference was not statistically significant. These results indicate that RIT and MA have similar staining susceptibility, with RIT demonstrating slightly lower surface roughness than MA.
3. A moderate positive correlation was observed in this thermocycling model, indicating that surface roughness influences color changes in this study, with an r_s_ value of 0.577. Although surface roughness is a significant determinant in the external staining of dental restorations, it is crucial to recognize that it is not the sole factor influencing this phenomenon. Other contributing variables, as previously discussed, interact synergistically to influence the extent of staining.

## Figures and Tables

**Table 1:** Descriptive data of median and ± median absolute deviation (MAD) of color values (L*, a*, b*).

Value	WSLs Creation		Treatment after WSLs					
			Resin Infiltration Group			Microabrasion Group		
	Sound Enamel (N = 78)	WSLs (N = 78)	Sound Enamel (N = 26)	WSLs (N = 26)	After Treatment (N = 26)	Sound Enamel (N = 26)	WSLs (N = 26)	After Treatment (N = 26)
L	85.40 ± 3.50	58.005 ± 5.25 *	84.30 ± 2.85 ^a^	58.2 ± 4.65 ^b^	86.30 ± 2.5 ^a^	86.30 ± 3.35 ^a^	55.15 ± 5.4 ^b^	91.75 ± 2.90 ^a^
a	3.3 ± 1.30	7.1 ± 1.2 *	3.75 ± 1.3 ^a^	7.6 ± 1.2 ^b^	2.90 ± 1.15 ^a^	3.25 ± 0.8 ^a^	7.7 ± 0.95 ^b^	2.45 ± 1.05 ^a^
b	39.75 ± 2.45	36.95 ± 1.85 *	41.20 ± 2.1 ^a^	37.7 ± 1.7 ^b^	37.60 ± 2.6 ^b^	38.8 ± 2.15 ^a^	36.3 ± 1.25 ^b^	39.85 ± 2.50 ^a^

* Significant differences between sound enamel and after WSL creation, using the Wilcoxon signed-ranked test (*p* < 0.05). ^a, b^ Different lower letters in the same row indicate significant differences among steps in each treatment, using the Friedman test (*p* < 0.05).

**Table 2:** Descriptive data of median and ± median absolute deviation (MAD) of surface roughness (R_a_, µm).

Timeline	Group					
	Artificial Saliva (Control) (N = 39)			Thermocycling in Red Wine (Staining) (N = 39)		
	1 Untreated WSLs (N = 13)	2 RI (N = 13)	3 MA (N = 13)	4 Untreated WSLs (N = 13)	5 RI (N = 13)	6 MA (N = 13)
Baseline	0.22 ± 0.05 ^a^	0.20 ± 0.07 ^ac^	0.18 ± 0.04 ^ac^	0.15 ± 0.05 ^a^	0.18 ± 0.05 ^a^	0.18 ± 0.05 ^ac^
Artificial WSLs	0.35 ± 0.09 ^b^	0.35 ± 0.06 ^b^	0.33 ± 0.11 ^bc^	0.30 ± 0.07 ^b^	0.29 ± 0.05 ^b^	0.51 ± 0.12 ^ad^
Treatments		0.23 ± 0.05 ^ab^	0.14 ± 0.05 ^a^		0.19 ± 0.08 ^a^	0.09 ± 0.01 ^c^
Immersion	0.30 ± 0.09 ^ab^	0.08 ± 0.02 ^c^	0.09 ± 0.02 ^a^	0.61 ± 0.18 ^b^	0.15 ± 0.03 ^a^	0.55 ± 0.09 ^bd^

^a, b, c, d^ Different lower letters in the same column indicate significant differences among the group for R_a_, using the Friedman test (*p* < 0.05).

**Table 3:** Median and ± median absolute deviation (MAD) data of the color change (∆E) between treatments after red wine thermocycling and surface roughness (R_a_) after red wine thermocycling.

Group		∆E	R_a_ (µm)
Control (N = 39)	1. untreated WSLs (N = 13)	2.77 ± 0.58 ^b^	0.30 ± 0.09 ^bc^
	2. resin infiltration (N = 13)	1.38 ± 0.31 ^b^	0.08 ± 0.02 ^a^
	3. microabrasion (N = 13)	1.95 ± 0.63 ^b^	0.09 ± 0.02 ^a^
Staining (N = 39)	4. untreated WSLs (N = 13)	N/A	0.61 ± 0.18 ^c^
	5. resin infiltration (N = 13)	31.40 ± 4.89 ^a^	0.15 ± 0.03 ^ab^
	6. microabrasion (N = 13)	43.94 ± 3.57 ^a^	0.55 ± 0.09 ^bc^

^a, b, c^ Different lower letters in the same column indicate significant differences among the group for ∆E and R_a_ (*p* < 0.05). Abbreviations: N/A: not applicable.

## Data Availability

The original contributions presented in the study are included in the article, further inquiries can be directed to the corresponding author.
